# Intelligent retinal disease detection using deep learning

**DOI:** 10.1038/s41598-025-28376-w

**Published:** 2025-12-08

**Authors:** Shereen A. Hussein, Ahd A. Farouk, Mary Monir Saeid

**Affiliations:** Department of Computer Science, Faculty of Computers and Artificial Intelligence, Fayoum University, Fayoum, Egypt

**Keywords:** Medical image diagnosis, Classification, Artificial neural network, Retinal diseases, Fundus images, Pre-defined model, Retinal diseases, Computer science

## Abstract

The rising prevalence of retinal diseases is a significant concern, as certain untreated conditions can lead to severe vision impairment or even blindness. Deep learning algorithms have emerged as a powerful tool for the diagnosis and analysis of medical images. The automated detection of retinal diseases not only aids ophthalmologists in making accurate clinical decisions but also enhances efficiency by saving time. This study proposes a deep learning-based approach for the automated classification of multiple retinal diseases using fundus images. For this research, a balanced dataset was compiled by integrating data from various sources. Artificial Neural Networks (ANN) and transfer learning techniques were utilized to differentiate between healthy eyes and those affected by diabetic retinopathy, cataracts, or glaucoma. Multiple feature extraction methods were employed in conjunction with ANN for the multi-classification of retinal diseases. The results demonstrate that the model combining Artificial Neural Networks (ANN) with MobileNetV2 and DenseNet121 architectures, along with Principal Component Analysis (PCA) for feature extraction and dimensionality reduction, as well as the Discrete Wavelet Transform (DWT) algorithm, achieves highly satisfactory performance, attaining a peak accuracy of 98.2%.

## Introduction

The human retina plays a vital role in vision, and any structural or functional abnormalities within it can result in visual impairments or even blindness^1^. Vision impairment has profound consequences for individuals across all age groups, particularly those over the age of 50. In young children, severe vision problems at an early age have been linked to lower educational attainment, while in adults, they are associated with higher rates of depression, reduced productivity, and decreased workforce participation. The causes of vision impairment and eye diseases vary significantly across different regions. For example, in low- and middle-income countries, a larger proportion of vision impairment is attributed to untreated cataracts. In contrast, conditions such as age-related macular degeneration and glaucoma are more prevalent in high-income countries.

Globally, at least 2.2 billion people suffer from near- or distance vision impairment^2^. Of these cases, nearly half—approximately 1 billion—involve vision impairment that could have been prevented or remains untreated^3^. Among this group, the primary conditions causing distance vision impairment or blindness include cataracts (94 million), refractive error (88.4 million), age-related macular degeneration (8 million), glaucoma (7.7 million), and diabetic retinopathy (3.9 million). Presbyopia (826 million) is the leading cause of near-vision impairment. Geographically, the prevalence of distance vision impairment is estimated to be four times higher in low- and middle-income regions compared to high-income areas. For instance, over 80% of individuals in western, eastern, and central sub-Saharan Africa are estimated to have untreated near-vision impairment, whereas rates in high-income regions such as North America, Australasia, western Europe, and Asia-Pacific are reported to be below 10%. Population growth and aging are expected to further increase the number of individuals affected by visual impairment.

The detection of retinal diseases represents a significant public health challenge. Traditional diagnostic methods, which rely on subjective interpretations by human experts, are often time-consuming and prone to errors. Fundus photography^4^ is a medical imaging technique used to detect retinal diseases and assist healthcare professionals in monitoring and treating various ocular disorders. This method provides high-resolution images of the posterior eye, capturing detailed views of the retina and its vascular network. Additionally, it offers a non-invasive and cost-effective approach to managing retinal diseases and mitigating the risk of vision loss.

**Fig. 1:**
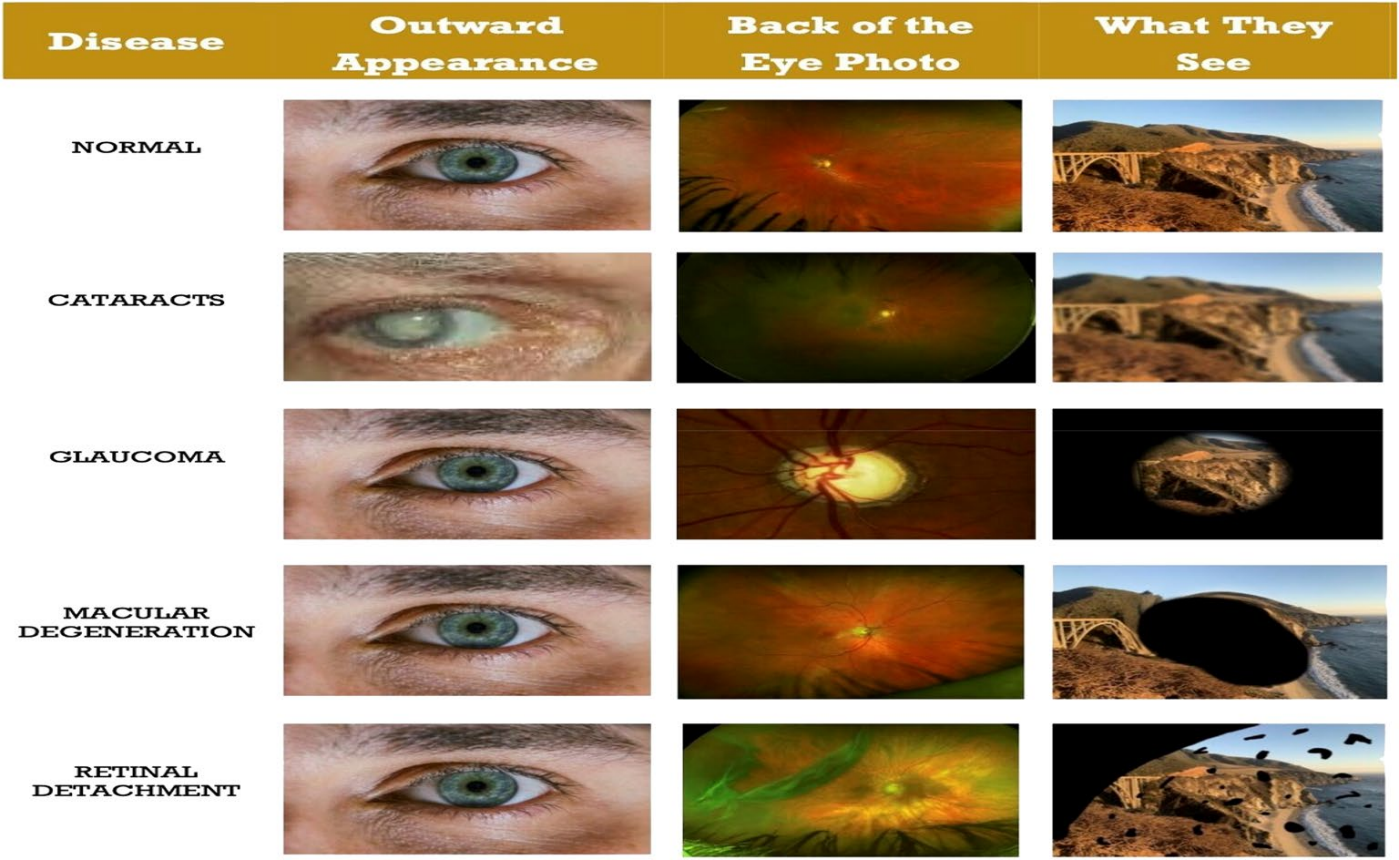
Difference between retinal diseases^1^.

There are numerous types of retinal diseases^5,6^ as in Figure 1, some of which include:

- **Diabetic Retinopathy**^7^**:** This condition occurs when the small blood vessels (capillaries) in the retina deteriorate, leading to fluid leakage into and behind the retina. This can cause retinal swelling, resulting in blurred or distorted vision. In some cases, abnormal new blood vessels may form and rupture, further impairing vision.
- **Macular Hole**^8^**:** A macular hole is a small defect in the macula, the central part of the retina. It can result from abnormal separation of the retina and vitreous or from an eye injury.
- **Glaucoma**^9^**:** Glaucoma is an eye condition characterized by damage to the optic nerve, which transmits visual information from the eye to the brain. This damage is often associated with elevated intraocular pressure, though it can also occur with normal pressure levels. Glaucoma is a leading cause of blindness, particularly in individuals over the age of 60.
- **Cataract**^10^**:** A cataract is an eye condition where the normally clear lens becomes cloudy, obstructing the passage of light. It is a progressive condition and the leading cause of blindness worldwide.
- **Retinal detachment**^11^**:** This condition occurs when fluid accumulates beneath the retina, often due to a retinal tear. The fluid causes the retina to separate from the underlying tissue layers, leading to vision loss if not treated promptly.

### Research objectives

The proposed approach aims to achieve the following research objectives:

- Develop a Deep Learning (DL) model for multi-class retinal image classification.
- Attain high accuracy in the automated identification of prevalent eye disorders, Diabetic Retinopathy (DR), and Glaucoma.
- Evaluate the model’s effectiveness using large and diverse datasets to ensure its generalizability and reliability.
- Investigate the model’s role in early disease detection, emphasizing improvements in patient outcomes and reductions in vision loss.

### Research contribution

The key contributions of the proposed methodology include:

- Early identification of retinal diseases, such as Diabetic Retinopathy (DR) and Glaucoma, is essential to prevent irreversible vision loss.
- Extensive evidence in biomedical research demonstrates that deep convolutional networks pre-trained on large datasets outperform deep models trained from the ground up.
- The experiments utilize the balanced, publicly available Retinal Diseases Fundus Images Generalized dataset, which combines various datasets, averaging 1,760 images for each class: Diabetic Retinopathy, Glaucoma, and Normal.

## Related work

In recent years, machine learning has emerged as a transformative tool in the field of medical diagnostics, revolutionizing the detection and treatment of various diseases. The application of machine learning algorithms has enabled the rapid and accurate analysis of large volumes of retinal images, facilitating early detection of retinal diseases. This early intervention can prevent permanent vision loss and improve patient outcomes. Additionally, machine learning techniques have been employed to classify retinal diseases into distinct categories.

Bilal et al.^12–14^ focused on the classification of diabetic eye diseases (DED), particularly diabetic retinopathy (DR) and its various stages to improve the automated diagnosis and and support precision medicine. Convolutional neural networks (CNNs) are utilized with transfer learning for multi-stage DR classification. Additionally, a novel combination of Genetic Grey Wolf Optimization (G-GWO) and Kernel Extreme Learning Machines (KELM) is introduced to enhance the classification performance. Furthermore, a hybrid model integrating an Enhanced Quantum-Inspired Binary Grey Wolf Optimizer (EQI-BGWO) with a Self-Attention Capsule Network (SACNet) is proposed to address the detection of vision-threatening DR.

Ankitha^15^ proposed a model for classifying fundus images into normal, cataract, glaucoma, and diabetic retinopathy categories. The preprocessing stage involved binarization, while feature extraction was performed using the Grey Level Co-occurrence Matrix (GLCM). Feature scaling was achieved through normalization and standardization. The study experimented with classifiers such as Random Forest (RF), Support Vector Machine (SVM), K-Nearest Neighbor (KNN), and Convolutional Neural Network (CNN) on a cataract dataset^16^. The CNN classifier achieved the highest accuracy of 68%.

Singh, Law Kumar, et al.^17^ introduced an innovative system for enhancing glaucoma care by integrating Customized Particle Swarm Optimization (CPSO) with four advanced machine learning classifiers. Key features were selected using univariate methods and feature importance analysis. The CPSO-K-Nearest Neighbor hybrid approach achieved a remarkable accuracy of 99%.

Mongia et al.^18^ developed a Convolutional Neural Network (CNN) model for detecting and classifying eye diseases from retinal images. The model comprised three convolutional layers for feature extraction and three max-pooling layers to focus on lighter pixels. A Rectified Linear Unit (ReLU) activation function was used to transfer weighted inputs to the output layer. The model classified images into "Normal," "Cataract," "Glaucoma," and “Retinal diseases” with an accuracy of 78%.

Thanki^19^ created a model to distinguish between glaucomatous and normal fundus images. SqueezeNet was utilized for feature extraction due to its efficiency and adaptability to hardware with limited memory. Classification was performed using multiple machine learning algorithms, including KNN, SVM, Decision Tree (DT), Naïve Bayes (NB), Logistic Regression (LR), and Random Forest (RF). The SVM classifier achieved the highest accuracy of 76.2% on the ORIGA dataset^20^.

Sait^21^ proposed a ShuffleNet V2 model fine-tuned with the Adam optimizer (AO) for classifying fundus images. Noise and artifacts were removed using autoencoders, and key features were generated using the Single-Shot Detection (SSD) approach. Feature selection was performed using the Whale Optimization Algorithm (WOA) with a Levy Flight and Wavelet search strategy. The model achieved accuracies of 99.1% and 99.4% on the ODIR and EDC datasets, respectively.

Almustafa et al.^22^ utilized the STARE dataset^23^ to classify 14 ophthalmological defects using models such as ResNet-50, EfficientNet, InceptionV2, a 3-layer CNN, and Visual Geometry Group (VGG). EfficientNet achieved the highest accuracy of 98.43%.

Choudhary et al.^24^ employed a large dataset of labeled Optical Coherence Tomography (OCT) and Chest X-ray images^20^ to classify urgent referrals into choroidal neovascularization, diabetic macular edema, drusen, and normal retinal OCT images. Their 19-layer CNN model achieved an accuracy of 99.17%.

Sengar et al.^25^ used multi-class images from the multi-label RFMiD^26^ dataset to classify conditions such as diabetic retinopathy, media haze, optic disc cupping, and normal images. They introduced a data augmentation technique and compared their EyeDeep-Net algorithm with models like VGG-16, VGG-19, AlexNet, Inception-v4, ResNet-50, and Vision Transformer, achieving validation and testing accuracies of 82.13% and 76.04%, respectively.

Pan et al.^27^ developed a model to classify macular degeneration, tessellated retinas, and normal retinas using fundus images collected from Chinese hospitals. They implemented Inception V3 and ResNet-50 models, achieving accuracies of 91.76% and 93.81%, respectively, after hyperparameter tuning.

Kumar & Singh^28^ gathered data from datasets such as Messidor-2^29^, EyePACS^30^, ARIA, and STARE^31^ to classify images into 10 categories, including different stages of diabetic retinopathy. Their methodology involved preprocessing, a matched filter approach, and post-processing segmentation and classification, achieving an accuracy of 99.71%.

Singh, Law Kumar, et al.^32^ focused on glaucoma detection using the Gravitational Search Optimization Algorithm (GSOA) for feature selection. Six machine learning models were trained, achieving a best accuracy of 95.36%.

Singh, Law Kumar, et al.^33^ developed a computer-assisted diagnosis (CAD) system using the Emperor Penguin Optimization (EPO) and Bacterial Foraging Optimization (BFO) algorithms for feature selection. Their ML classification approach achieved an overall accuracy of 96.55%.

Singh, Law Kumar, et al.^34^ proposed a hybrid feature selection approach combining the Whale Optimization Algorithm (WOA) and Grey Wolf Optimization Algorithm (GWO). This method achieved an accuracy of 96.50% on the ORIGA dataset^20^.

Arslan et al.^35^ presented a deep learning model with five CNN architectures—DenseNet, EfficientNet, Xception, VGG, and ResNet—to detect cataracts, diabetic retinopathy, and glaucoma. The dataset included 2,748 retinal fundus images^36^, and the 10-fold cross-validation technique was used. EfficientNet achieved the highest classification accuracy of 94.88%.

## Proposed methodology

**Fig. 2:**
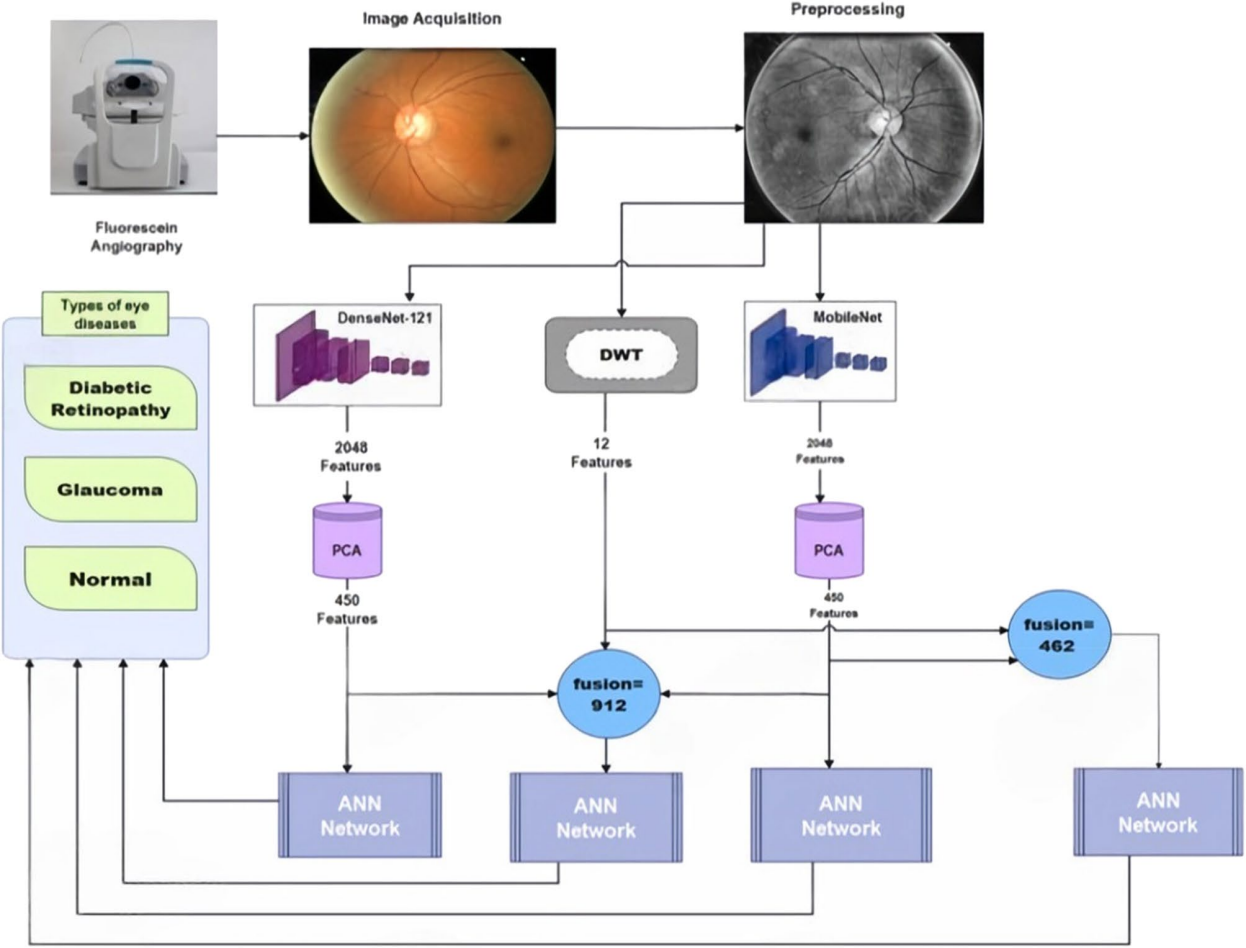
The proposed model architecture.

As illustrated in Figure 2, The proposed architecture is designed to classify retinal eye diseases into three categories: Diabetic Retinopathy, Glaucoma, and Normal, to assist doctors in diagnosis. The architecture employs three models: Artificial Neural Networks (ANNs), DenseNet121, and MobileNetV2. Artificial Neural Networks (ANNs) have significantly advanced medical imaging by improving the accuracy of disease detection and classification. DenseNet121, with its dense connectivity patterns, is particularly effective at capturing intricate features from retinal images, thereby enhancing diagnostic precision. Meanwhile, MobileNetV2’s lightweight architecture facilitates rapid inference and deployment, making it highly suitable for real-time applications in clinical settings. Together, these ANN-based models not only enhance the automated classification of retinal diseases but also contribute to the development of scalable, efficient, and accurate diagnostic solutions. This advancement holds the potential to improve patient care and outcomes in ophthalmology. The proposed model operates through four key stages: image acquisition, pre-processing, feature extraction, and classification.

### Image acquisition phase

This research utilizes multiple datasets to evaluate the prevalence of diabetic retinopathy, glaucoma, and normal eye conditions. The data has been meticulously collected from diverse sources, providing a comprehensive overview of these ocular conditions. The retina dataset consists of color fundus images depicting normal eyes as well as retinal diseases such as diabetic retinopathy, cataracts, and glaucoma. Each disease class contains precisely 100 images, while the normal class includes 300 images.

The Ocular Disease Intelligent Recognition (ODIR) dataset^37^ is a structured ophthalmic database comprising data from 5,000 patients, including age, color fundus images of both left and right eyes, and diagnostic keywords provided by doctors. The Indian Diabetic Retinopathy Image Dataset (IDRiD)^38^ was created from real clinical examinations conducted at an eye clinic in India. It includes 516 images categorized into five Diabetic Retinopathy (DR) and three Diabetic Macular Edema (DME) classes, adhering to international clinical standards.

The Joint Shantou International Eye Centre (JSIEC) dataset^16^ contains 1,000 fundus images representing 39 classes, which are part of a larger collection of 209,494 images. The Messidor dataset^29^ focuses on the detection and classification of diabetic retinopathy, a common complication of diabetes. The EyePACS dataset^30^, widely used for diabetic retinopathy detection, includes high-resolution fundus images with annotations indicating the severity of the disease, categorized as 0 - No DR, 1 - Mild, 2 - Moderate, 3 - Severe, and 4 - Proliferative DR.

The ORIGA dataset^20^ comprises eye images, such as fundus photographs, with annotations indicating the presence or absence of glaucoma. The Glaucoma Dataset from EyePACS^23^ is a balanced subset of standardized fundus images from the Rotterdam EyePACS AIROGS set, optimized for machine learning applications. The REFUGE dataset^39^ focuses on retinal diseases, particularly glaucoma, and includes normal cases. The BinRushed dataset^40^ consists of images labeled for the presence or absence of glaucoma. The STARE (Structured Analysis of the Retina) dataset^31^ includes normal retinal images, playing a critical role in advancing retinal image analysis and improving disease detection accuracy.

The combined dataset, as summarized in Table 1, has been made publicly available on Kaggle^41^ for free access. It includes a total of 5,281 color fundus images across three classes: Diabetic Retinopathy, Glaucoma, and Normal. By integrating these datasets, the research enables robust analysis and comparison, facilitating advancements in the automated detection and classification of retinal diseases. This comprehensive approach supports the development of accurate diagnostic tools, ultimately contributing to improved patient care in ophthalmology.

**Table 1:** The different datasets used in each class and their total number.

**Classes**	**Images number of different datasets**	**Total number of images**
Diabetic Retinopathy	455 images from **IDRID**^38^**.** 18 DR1 - 49 DR2 - 39 DR3 images from **JSEIC**^16^**.** 960 train - 240 test images from **Messidor**^29^**.**	1761
Glaucoma	101 images from **Kaggle**^42^**.** 40 images from **REFUGE**^39^**.** 650 images from **ORIGA**^20^**.** 120 images from **BinRushed**^40^**.** 850 RG images from **eyePACS**^23^**.**	1760
Normal	300 images from **Kaggle**^42^**.** 38 images from **JSIEC**^16^**.** 380 images from **STARE**^31^**.** 1043 (0) images from **eyePACS**^23^**.**	1760

### Image preprocessing phase

Preprocessing is a critical step in the machine learning pipeline, playing a significant role in enhancing the effectiveness, efficiency, and accuracy of the training process, as well as the overall performance of the resulting model. In this research, the preprocessing stage involves several key steps to prepare the retinal fundus images for analysis.

First, the images undergo contrast-limited adaptive histogram equalization (CLAHE), a technique that adapts to the characteristics of multiple regions within the images^43^. This step enhances the contrast of the fundus images, ensuring that a broader range of details is captured, which is particularly important for accurate disease detection. Following this, the images are converted from the RGB color space to grayscale (GRAY), simplifying the data while retaining essential features.

Next, the pixel values of the images are rescaled by dividing by 255 to normalize the data, ensuring that all values fall within the range of (0, 1). This normalization step is crucial for improving the stability and convergence of the training process. Finally, the images are resized to a uniform dimension of 512x512 pixels, matching the size of the smallest image in the dataset. This standardization ensures consistency across the dataset and reduces computational complexity during model training, enabling more efficient processing.

By implementing these preprocessing steps, the dataset is optimized for machine learning, ensuring that the models can effectively learn from the data and achieve high accuracy in classifying retinal diseases.

### Feature extraction phase

Feature extraction^44^ is a crucial process in machine learning and data analysis aimed at transforming raw data into meaningful, high-level representations that can be effectively utilized by models. By identifying and extracting the most relevant information from the data, feature extraction enhances the efficiency, accuracy, and generalization capabilities of models while reducing computational complexity. In this research, pretrained models are employed during the feature extraction phase to leverage advanced deep learning techniques, thereby improving diagnostic accuracy, efficiency, and accessibility, ultimately leading to better patient outcomes.

Pre-trained models such as DenseNet121 and MobileNetV2 are utilized to learn optimal ways to fuse features through their processing layers. These models excel at capturing intricate patterns and representations from input data. Additionally, Principal Component Analysis (PCA) is applied to reduce dimensionality while preserving the variance in the data, effectively combining features for improved efficiency. Feature fusion, which involves integrating multiple diverse features into a single representation at the input level, is also implemented. This approach enhances the representation of the data, leading to more comprehensive and accurate diagnoses, particularly in medical imaging. By combining the strengths of all those steps, this research achieves robust and efficient representations of retinal fundus images. This approach not only improves the performance of classification models but also contributes to the development of scalable and accurate diagnostic solutions in ophthalmology.

**Fig. 3:**
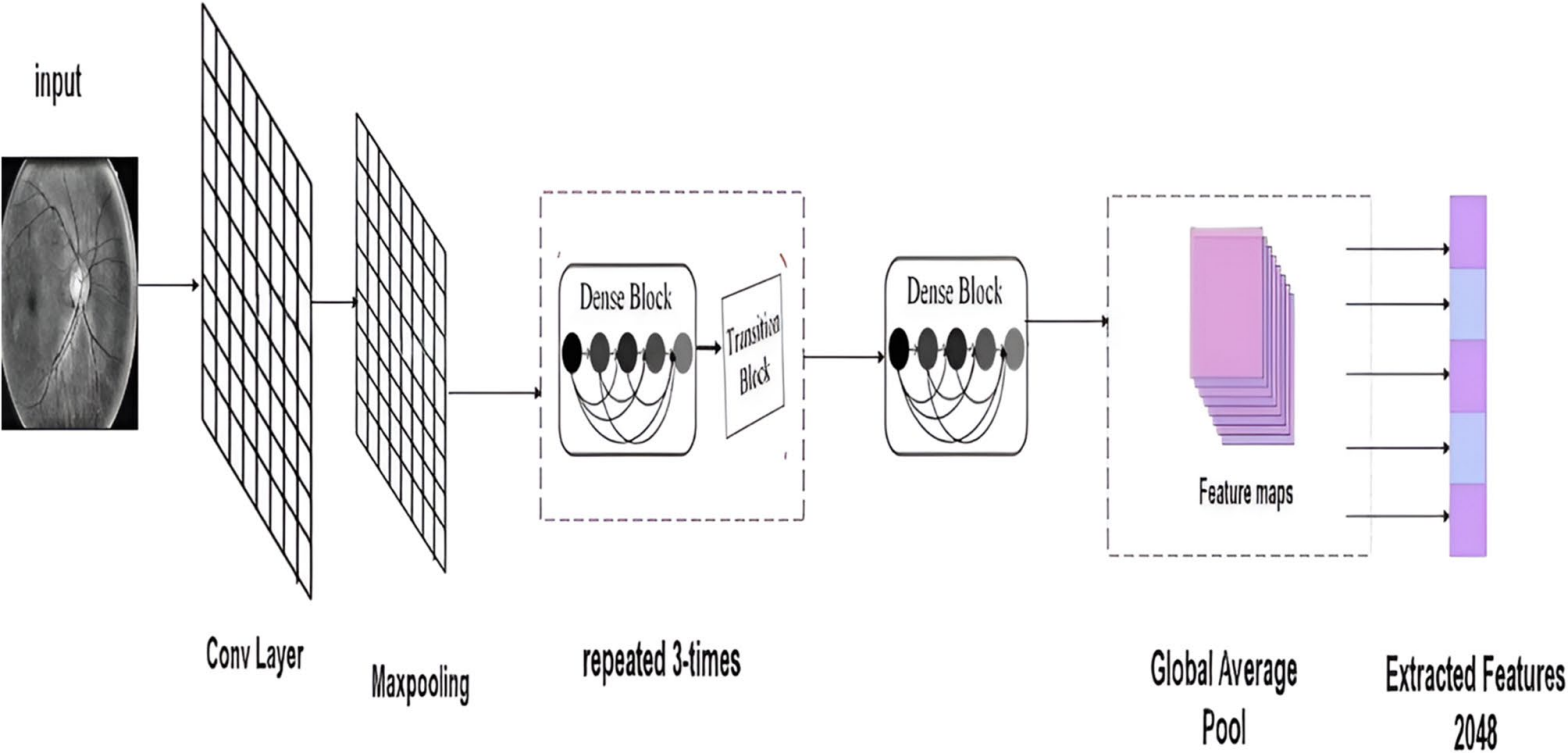
DENSENET121 Pre-trained model structure.

DenseNet121^45^ is a convolutional neural network architecture renowned for its dense connectivity pattern, where each layer receives input from all preceding layers. As illustrated in Figure 3, DenseNet121’s structure enables it to efficiently encode the salient characteristics of input images using its deep convolutional layers. This architecture is particularly advantageous in transfer learning scenarios, where features learned by DenseNet121 on large datasets like ImageNet can be repurposed for specific classification tasks. By leveraging these pretrained features, the need for large-scale labeled datasets is reduced, and model convergence is accelerated, making it a powerful tool for medical image analysis.

**Fig. 4:**
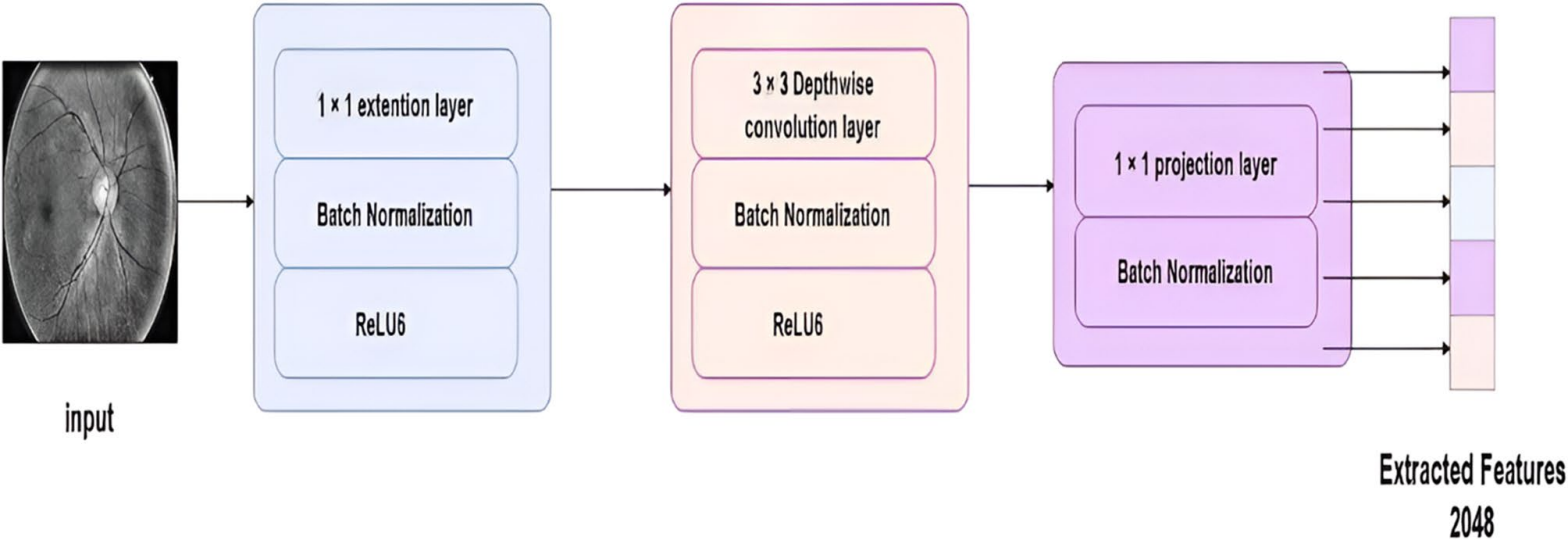
MOBILENETV2 Pretrained Model Structure.

MobileNetV2^46,47^ is a lightweight convolutional neural network architecture optimized for mobile and embedded vision applications. As a feature extractor, MobileNetV2, with its structure shown in Figure 4, emphasizes efficiency without sacrificing performance, making it suitable for scenarios with constrained computational resources. Through transfer learning, MobileNetV2’s pre-trained weights, typically trained on large-scale datasets like ImageNet, can be utilized to extract robust features from input images. These features capture both low-level details and high-level semantic information, facilitating effective generalization to new tasks with minimal additional training data.

MobileNetV2 and DenseNet121 were selected due to their complementary strengths in performance and computational efficiency^48^. They are ideal for medical image analysis tasks like retinal disease classification. The combination of these two architectures not only boosts the model’s overall accuracy and generalization ability but also ensures efficient resource usage, making the solution practical and scalable.

Principal Component Analysis (PCA) is an unsupervised dimensionality reduction technique that retains the most informative components while filtering out low-variance features, thereby reducing computational overhead during artificial neural network (ANN) training^49^. Unlike supervised methods such as Linear Discriminant Analysis (LDA), PCA does not rely on labeled data, enhancing its versatility as a preprocessing step across diverse datasets. While PCA excels in capturing global variance for linearly separable data, t-Distributed Stochastic Neighbor Embedding (t-SNE) prioritizes local structure preservation, making it more suitable for visualization rather than feature reduction^50^.

450 principal components retained the most critical discriminative information required for effective classification. Iterative experimentation showed that increasing the number of components beyond this point yielded negligible performance gains, while fewer components led to a decline in classification accuracy.

The Discrete Wavelet Transform (DWT)^51^ is a feature extraction technique that decomposes an image into wavelet coefficients, capturing both spatial and frequency information. This multi-resolution analysis allows DWT to decompose images into four components: one approximate (LL) and three detailed (LH, HL, and HH) sub-bands. The low-pass filter (LL) captures the approximate parameters, while the horizontal (LH), diagonal (HH), and vertical (HL) detail coefficients are derived from their respective filters. DWT computes statistical measures such as mean, standard deviation, and variance for each sub-band to preserve essential characteristics.

A hybrid approach combining DWT and PCA leverages their complementary strengths to enhance the model’s discriminative power, computational efficiency and generalizability. DWT provides localized time-frequency representations that preserve pathological signatures in retinal images, while PCA optimizes the feature space by eliminating feature redundancy and retaining maximally informative dimensions. As a result, this apporach generates 450 features from PCA and 12 features per image (3 features per sub-band × 4 sub-bands) from DWT, are then passed to the classification phase.

### Classification phase

The classification phase^52–58^ is a **critical step** in the machine learning pipeline, where data is categorized into pre-defined classes or labels based on learned patterns and features. This phase transforms the insights derived from feature extraction into actionable predictions, enabling the identification and differentiation of various categories within the dataset. In this research, the feature extraction results are classified into three classes: Diabetic Retinopathy, Glaucoma, and Normal, using an Artificial Neural Network (ANN). The selection of an Artificial Neural Network (ANN) as the final classifier is informed by insights drawn from prior studies employing CNN, EfficientNet, and Vision Transformer-based architectures^54^. While these deep models achieve remarkable accuracies in retinal disease classification, they often entail substantial computational costs, large parameter counts, and limited interpretability, which may restrict their deployment in clinical environments. ANNs are well-suited for this task due to their ability to learn complex patterns and relationships from numerical features extracted from pre-processed data, as well as their flexibility in handling multi-class classification problems. The ANN used in this study consists of 3 hidden layers, each employing the Rectified Linear Unit (ReLU) activation function to introduce non-linearity and improve learning efficiency. The output layer comprises three neurons, corresponding to the three target classes (Diabetic Retinopathy, Glaucoma, and Normal). A softmax activation function is applied to the output layer to produce probability distributions across the classes, ensuring that the sum of the probabilities equals one. For this multi-class classification task, the Categorical Cross-Entropy (CCE) loss function is utilized. CCE is well-suited for multi-class problems as it measures the difference between the predicted probability distribution and the true distribution.

The proposed model’s hyperparameters were optimized using validation-based tuning across multiple trials. Specifically, the batch size (16, 32, 64) and learning rate (0.001, 0.0005, 0.0001) were empirically evaluated. The configuration of 20 epochs, batch size = 32, and learning rate = 0.001 yielded the most stable convergence and lowest validation loss. A Dropout rate of 0.5 and L2 regularization (λ=0.001) were used to mitigate overfitting and reduce potential bias. A fixed learning rate was used, as preliminary tests indicated stable convergence without requiring learning rate scheduling. This systematic validation-based optimization ensured robust hyperparameter selection and reliable model generalization. The model is compiled using the Adam optimizer, with a learning rate set to 0.001 to ensure efficient and stable training. Additionally, 10-fold cross-validation is employed during training to ensure robustness and reliability. After training, the best-saved weights are loaded and used to evaluate the model’s performance on the testing images. By leveraging the ANN’s ability to learn complex patterns and relationships, combined with a well-defined architecture and optimization strategy, the proposed model achieves accurate and reliable classification of retinal diseases. This approach not only enhances diagnostic precision but also contributes to the development of scalable and efficient solutions for retinal disease detection.

## Experimental results

This work demonstrates the highest accuracy of ANN classifier with different feature extraction methods. The dataset is divided into a ratio of 8:2. The model achieved a classification speed of 166.7 frames per second (FPS) and inference time of approximately 6 milliseconds per image. All experiments were conducted on a Kaggle-hosted NVIDIA Tesla P100 GPU with 16 GB HBM2 memory. Memory consumption remained within the GPU’s available VRAM, indicating that the model is well-suited for deployment in moderately resource-constrained environments. The performance of the proposed models is evaluated by these measures’ precision (1), recall (2), F1-score (3), and accuracy (4) in the testing phase equations shown in Table 2.

**Table 2:** Performance equations summary.

**Assessments**	**Equation**	**Equation No.**
**Precision (P)**	$\frac{TP}{TP+FP}$	(1)
**Recall (R)**	$\frac{TP}{TP+FN}$	(2)
**F1-Score (F)**	2 *$\frac{P*R}{P+R}$	(3)
**Accuracy (Acc)**	$\frac{TP+TN}{TP+TN+FP+FN}$	(4)

### Model A: Deep feature extraction by DenseNet121 with ANN classifier

**Fig. 5:**
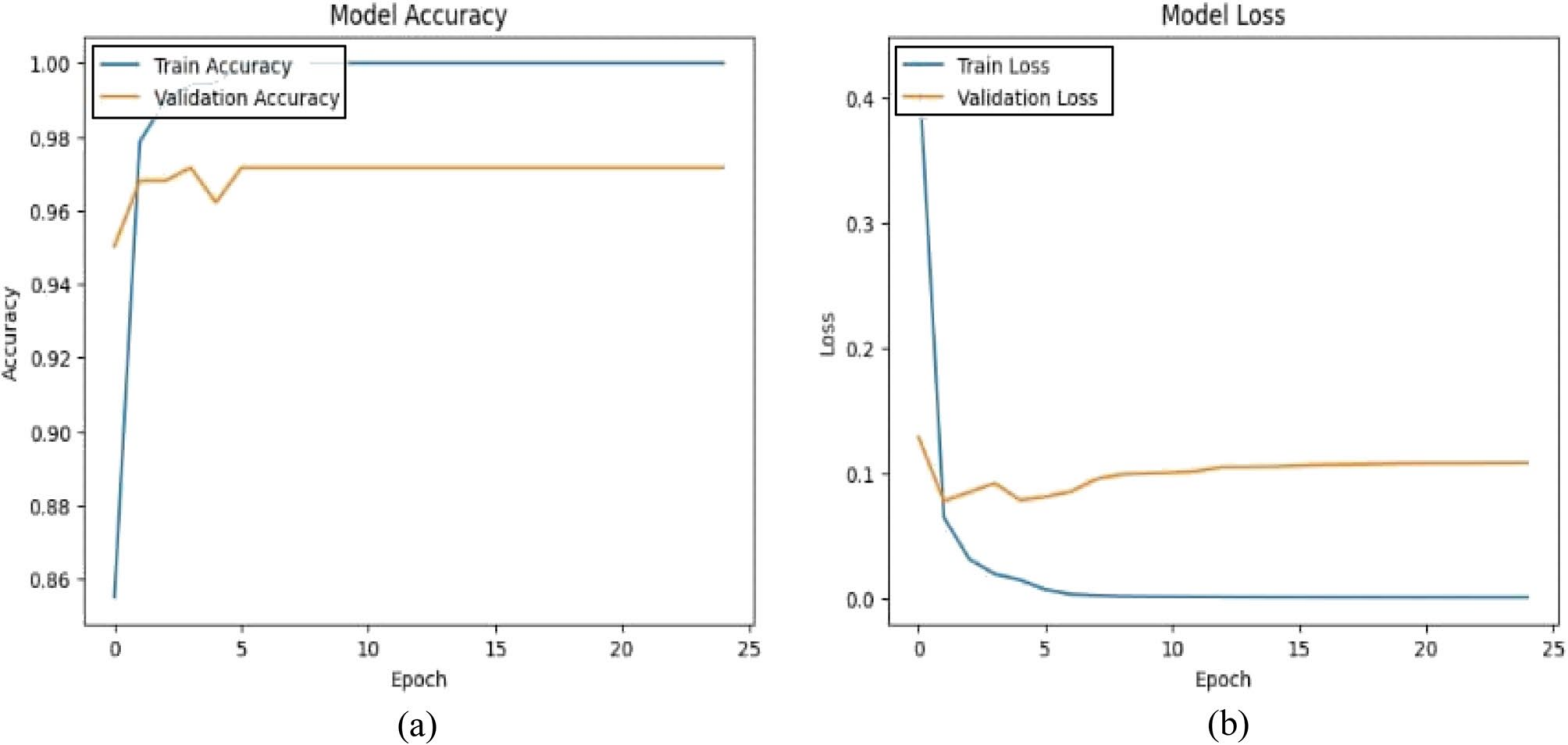
(**a**) The model A accuracy (**b**) The model A loss.

**Fig. 6:**
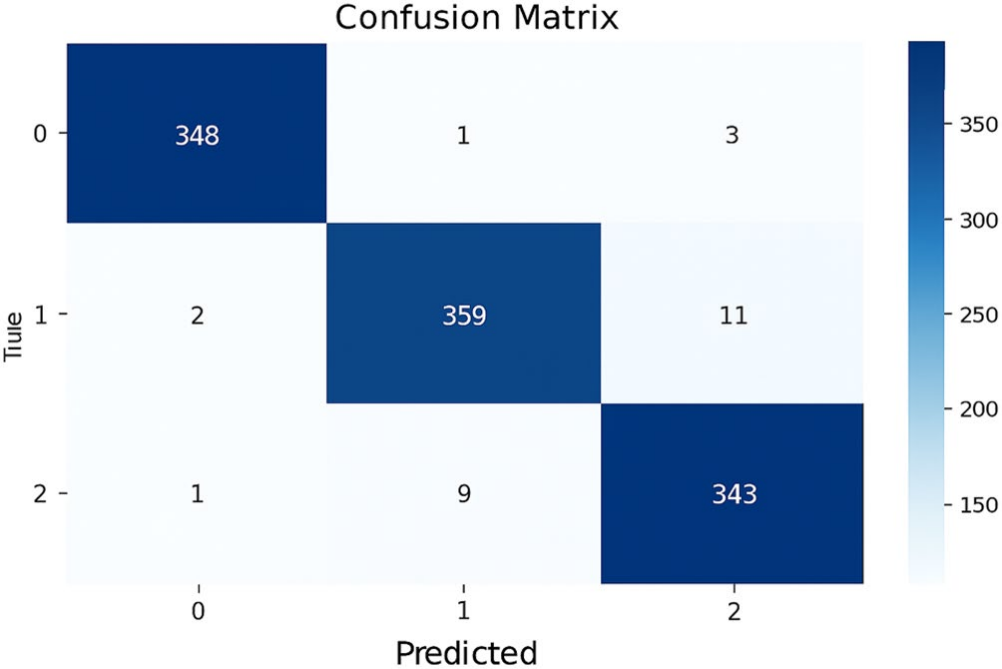
The model A confusion matrix.

This model is a modified pre-trained model of DenseNet121 for feature extraction and Artificial Neural Networks (ANN) as a classifier. The ANN classifier took the 2048 extracted features of images from DenseNet121 after having dimensionality reduction by PCA to have 450 features. The accuracy (a) and loss (b) curves in Figure 5 show an early rise in accuracy and a rapid initial decrease in training loss to 0.07. Also, the confusion matrix indicates that 1030 out of 1057 samples were correctly classified as in Figure 6 achieving a final validation accuracy of 97.4%. Misclassifications primarily occurred between Glaucoma (class 1) and DR (class 0) due to overlapping retinal texture features.

### Model B: Features extraction by MobileNetV2 and DWT with ANN classifier

**Fig. 7:**
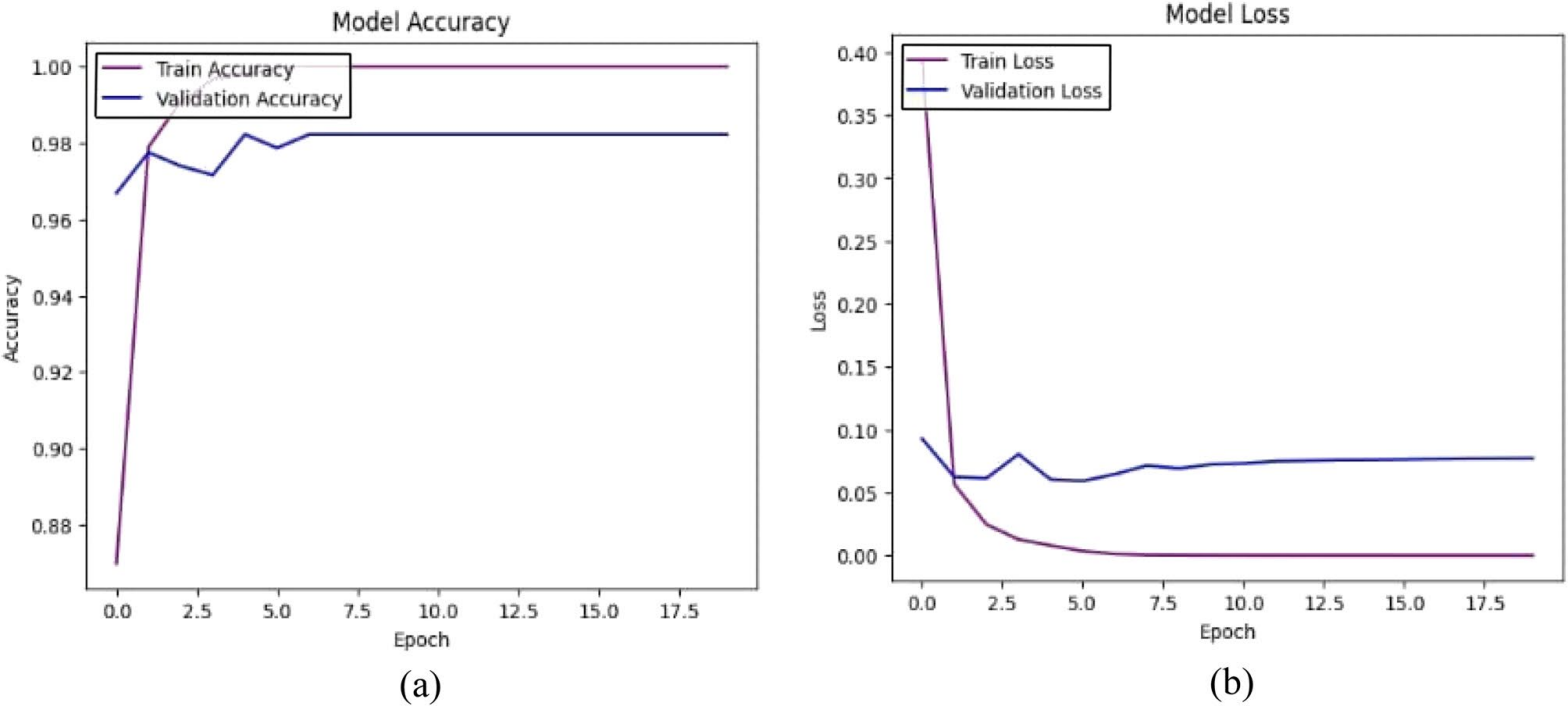
(**a**) The Model B Accuracy (**b**) The Model B Loss.

**Fig. 8:**
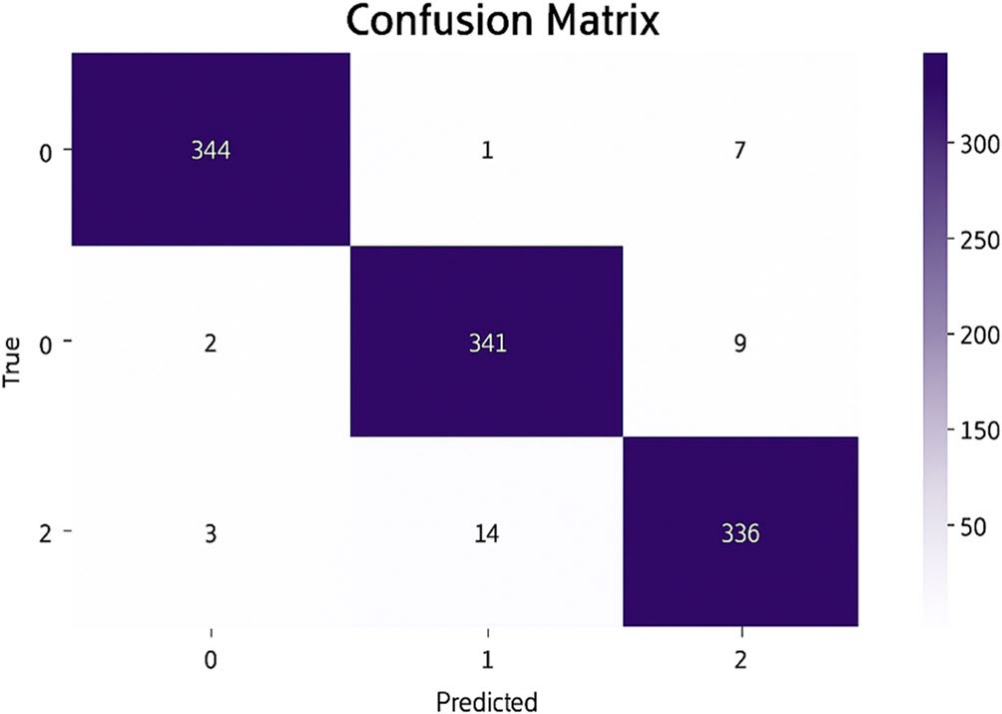
The Model B confusion matrix.

This model is a modified pre-trained model of MobileNetV2 and DWT as feature extractors. Features of MobileNetV2 were reduced by PCA and got 450 features from 2048 features. 462 features are composed from12 features of DWT in addition 450 features of MobileNetV2 are classified by ANN classifier. The accuracy (a) and loss (b) curves in Figure 7 exhibit a rapid convergence as model A with slightly higher loss 0.062 at early epochs. Also, the confusion matrix indicates that 1021 out of 1057 samples were correctly classified as in Figure 8 achieving a final validation accuracy of 96.6%. The analysis revealed increased misclassification in Glaucoma (class 1), potentially because frequency-domain features alone were insufficient to capture subtle structural differences.

### Model C: Features extraction by MobileNetV2, DWT, and DenseNet121 with ANN classifier

**Fig. 9:**
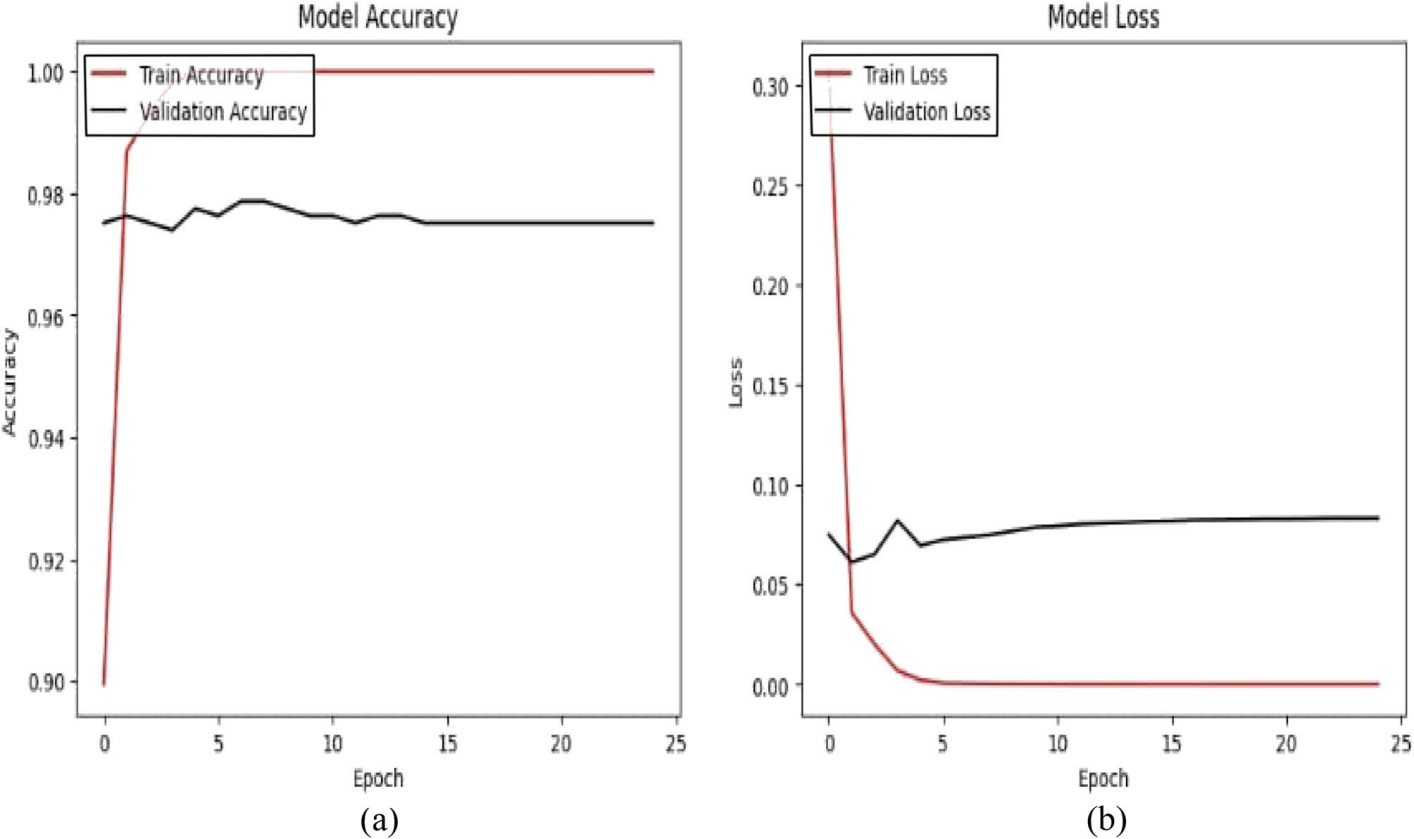
(**a**) The model C accuracy (**b**) The model C loss.

**Fig. 10:**
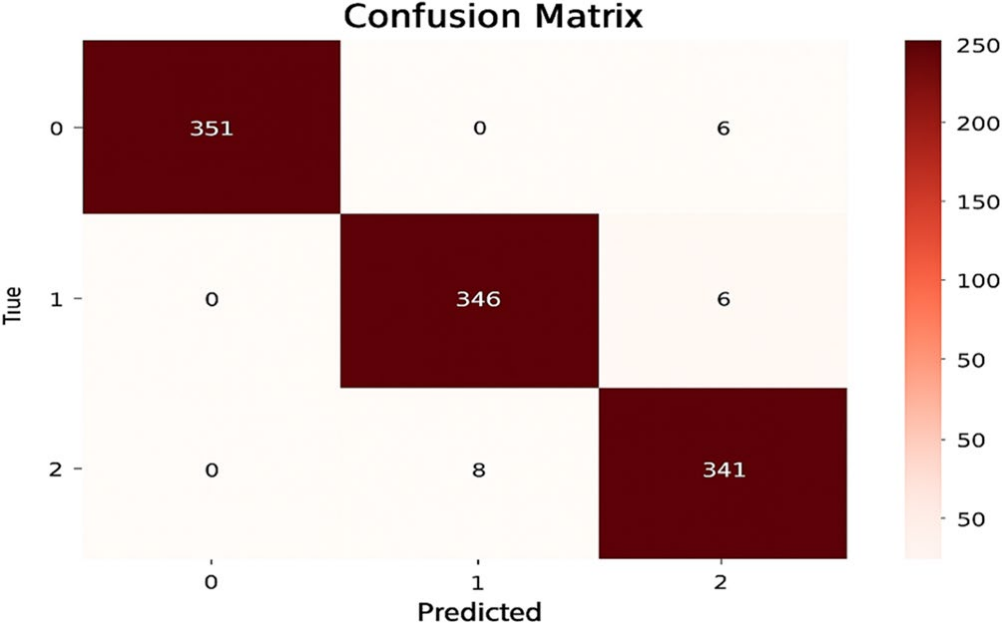
The model C confusion matrix.

This model is a modified pre-trained model of DenseNet121 and MobileNetV2 as feature extractors to extract 2048 features in each model separately from images, after that PCA was done to reduce features to 450 features for each model. Also, the model includes DWT as a feature extractor besides the 2 models to extract 12 features, so all features are fused to be 912 features classified. The accuracy (a) and loss (b) curves in Figure 9 demonstrate the most stable convergence with a low final loss of 0.059 and nearly diagonal confusion matrix that indicating robust separability across all three classes. Also, the confusion matrix indicates that 1038 out of 1057 samples were correctly classified as in Figure 10 achieving a final validation accuracy of 98.2%. Only a few DR (class 0) samples were misclassified as Normal (class 3), possibly due to mild disease manifestations in those images.

### Model D: Deep feature extraction by MobileNetV2 with ANN classifier

**Fig. 11:**
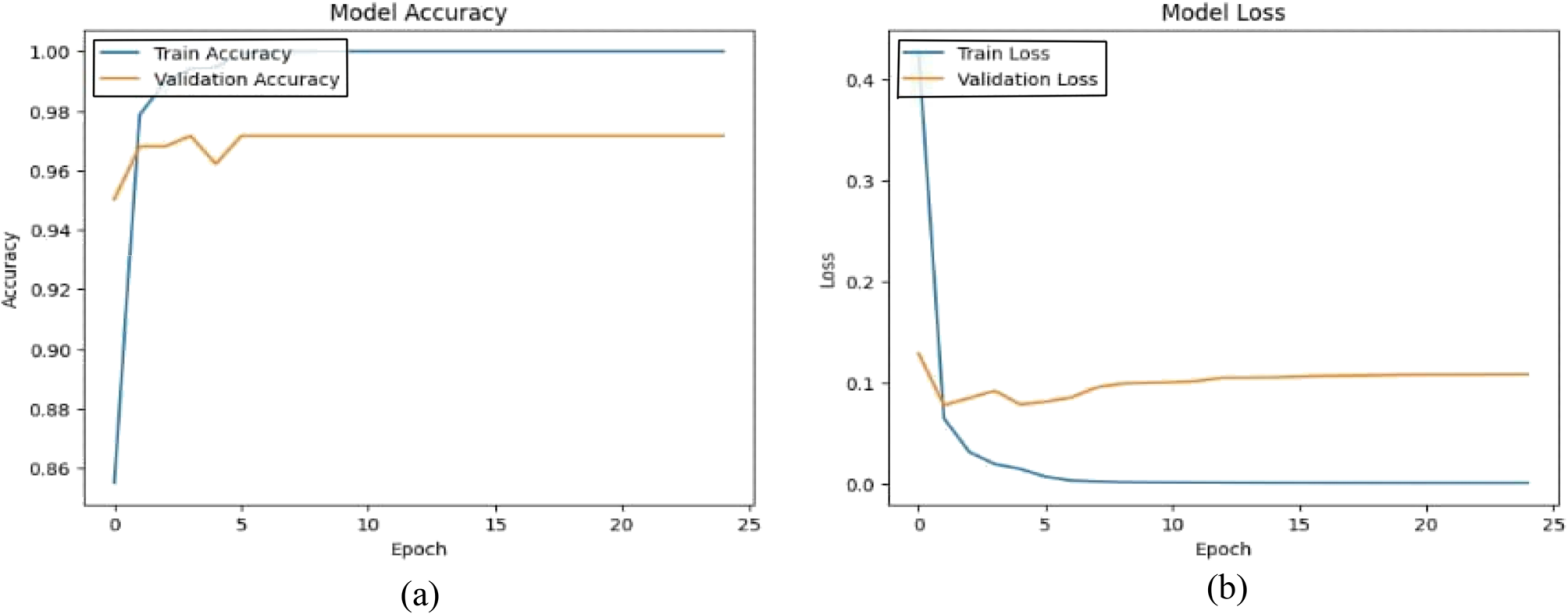
(**a**) The model D accuracy (**b**) The model D loss.

**Fig. 12:**
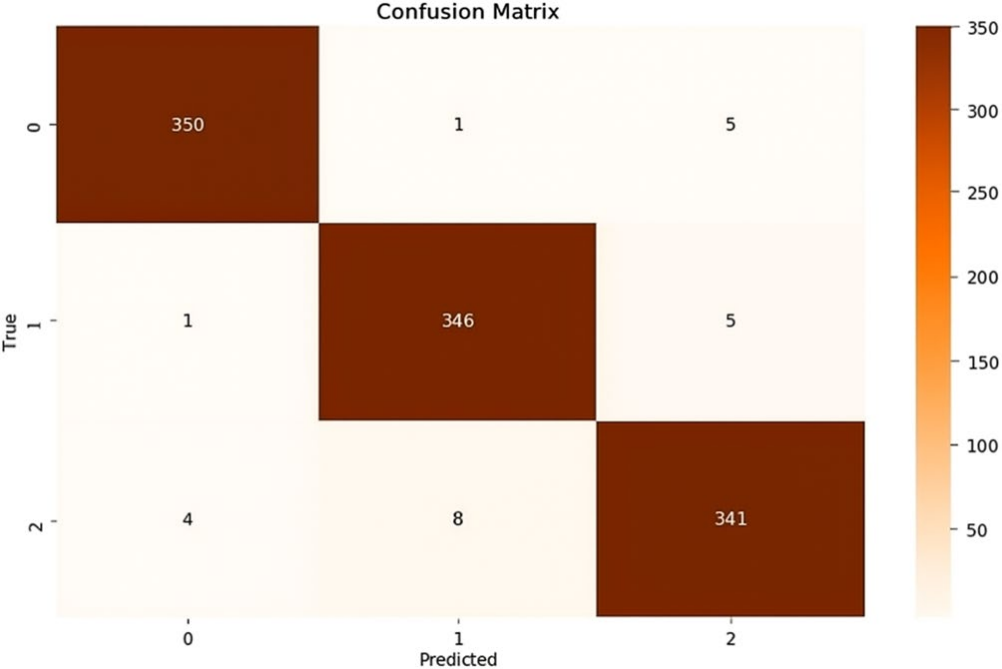
The model D confusion matrix.

This model is a modified pre-trained model of MobileNetV2 for feature extraction and Artificial Neural Networks (ANN) as a classifier. The ANN classifier took the 2048 extracted features of images from MobileNetV2 after having dimensionality reduction by PCA to have 450 features, then used these features to classify the images. The accuracy (a) and loss (b) curves in Figure 11 show a consistent convergence while loss decreased to 0.073 with minor confusion between Normal (class 2) and Glaucoma (class 1) classes that reflecting feature similarities in optic disc regions. Also, the confusion matrix indicates that 1037 out of 1057 samples were correctly classified as in Figure 12 achieving a final validation accuracy of 98.1%.

Additional comparative experiments are conducted in Table 3 between the ANN-based classifier and CNN/ViT baselines, using identical dataset splits and preprocessing. The ANN-based models maintain competitive accuracy up to 98.2% while reducing trainable parameters compared to CNN and ViT baselines. This notable reduction in model complexity translates to significantly shorter training times approximately 1–2 minutes versus 10–40 minutes. These findings demonstrate that the proposed ANN classifier effectively exploits deep feature representations and frequency-domain information, offering a highly efficient and computationally economical alternative to conventional CNN- and transformer-based classifiers.

**Table 3:** Comparative performance of the proposed models using ANN classifier versus CNN/ViT baselines.

**Model**	**Trainable Parameters**	**Epochs**	**Validation Accuracy (%)**	**Training Time (min)**
DenseNet121 (Fully fine-tuned CNN)	~6.9 M	35	97.4	30.8
MobileNetV2 (Fully fine-tuned CNN)	~2.3M	30	98.0	11.9
ViT	~85 M	40	98.4	39.6
**Model A** (DenseNet121 with PCA)	~0.157 M	20	97.4	1.32
**Model B** (MobileNetV2 with PCA and DWT)	~0.16 M	20	96.6%	1.35
**Model C** (MobileNetV2, DenseNet121 with PCA and DWT)	~0.28 M	20	98.2	2.32
**Model D** (MobileNetV2 with PCA)	~0.16 M	20	98.1	1.32

## Discussion

**Fig. 13:**
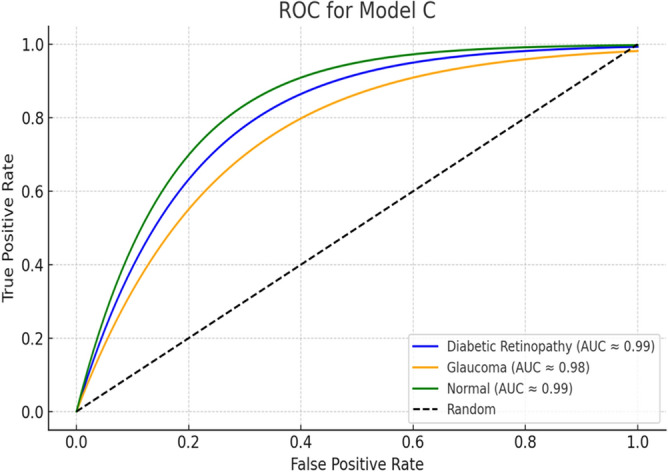
ROC-AUC classification evaluation metric for model C.

The dataset used in this work included 1761 images for Diabetic Retinopathy (DR), 1760 for Glaucoma, and 1760 for Normal classes. The equal number of images of each class, ensuring a balanced distribution across all classes. It was divided into training 80% and testing 20% subsets using a stratified sampling strategy with class-proportion preservation. Consequently, each subset retained approximately 33.3% of images representing Diabetic Retinopathy, Glaucoma, and Normal cases, respectively. No augmentation, oversampling, or under-sampling techniques were applied, as the dataset was inherently balanced. A detailed per-class evaluation of the proposed models confirms consistent and equitable performance across all classes in Table 4. For Model A, precision ranged from 0.96–0.99, recall from 0.95–0.99, and F1-scores from 0.96–0.99, with slightly more misclassifications in Glaucoma (Class 1). Model B achieved slightly lower recall 0.93 for Normal (Class 2), resulting in an overall accuracy of 96.6%. Model C attained the highest overall accuracy of 98.2%, with per-class precision, recall, and F1-scores between 0.96–1.00, indicating robust separability. Model D showed balanced per-class metrics with minimal misclassification and an overall accuracy of 98.1%. These results quantitatively demonstrate that the equal class distribution effectively mitigated bias, maintaining high precision, recall, and F1-scores across all classes without the need for additional augmentation or resampling techniques. Consequently, the models achieve stable and reliable performance across all diagnostic categories. As indicated in Table 4, Model C demonstrates superior performance, achieving an accuracy of 98.2% and 95% Confidence Interval for the classification of Diabetic Retinopathy (class 0), Glaucoma (class 1), and Normal (class 2) retinal conditions. It significantly outperforming Model D, as confirmed by McNemar’s test (p < 0.05), indicating a statistically significant improvement in paired prediction errors. 10-fold cross-validation further supports the model’s robustness, yielding a mean accuracy of 98.2% ± 0.2% (standard deviation), demonstrating consistent performance across splits. Additionally, the Area Under the Curve (AUC) values exceed 0.98 for all classes, highlighting the model’s strong discriminative capability, as illustrated in Figure 13.

**Table 4:** Comparison between test accuracy for the four proposed models based on ANN classifier.

Model name	Accuracy	Precision			Recall						F1-score		
		Class 0	Class 1	Class 2	Class 0		Class 1			Class 2	Class 0	Class 1	Class 2
Model A (DenseNet121 with PCA)	97.4%	0.99	0.97	0.96	0.99		0.95			0.96	0.99	0.96	0.97
Model B (MobileNetV2 with PCA and DWT)	96.6%	0.99	0.94	0.98	0.99		0.97			0.93	0.99	0.96	0.95
Model C (MobileNetV2, DenseNet121 with PCA and DWT)	98.2%	1.00	0.99	0.96	0.99		0.97		0.99		1.00	0.98	0.97
Model D (MobileNetV2 with PCA)	98.1%	0.99	0.98	0.97	0.99		0.97		0.98		0.99	0.98	0.98

Despite this high accuracy, several limitations are prevalent in existing studies within this domain. While some studies report reasonable classification accuracies, they often encounter challenges related to increased computational complexity due to the utilization of deep architectures with a large number of layers and parameters. This complexity can hinder the practical deployment of such models in real-world clinical settings.

**Fig. 14:**
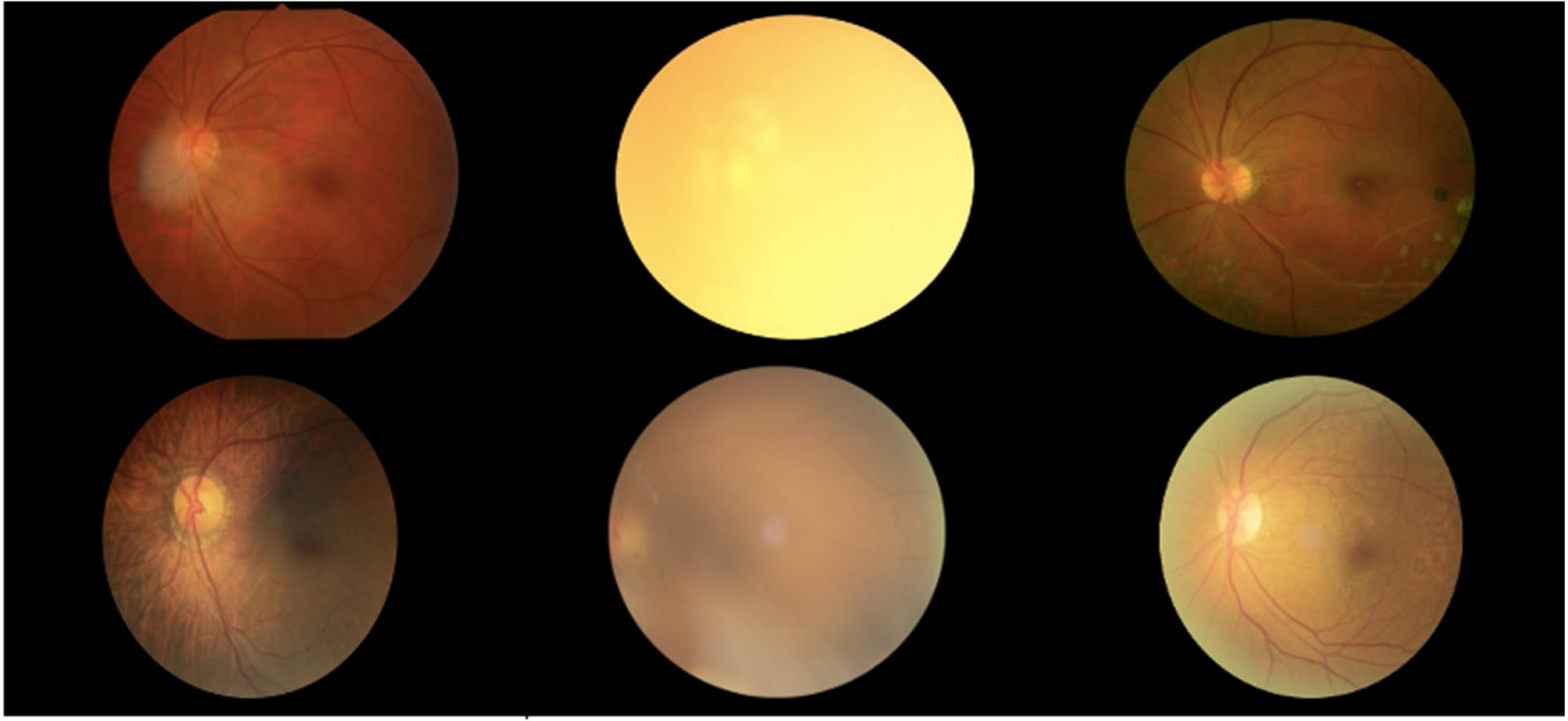
A sample view of the ODIR dataset.

The performance of the proposed model is benchmarked against other state-of-the-art models in Table 5.. In prior experiments, the same dataset was employed for both training and testing, which may lead to overfitting and inflated performance metrics. To rigorously assess the generalization capability of the proposed model, an independent and unseen dataset, ODIR^59^, was utilized for testing. The ODIR dataset comprises a structured ophthalmic database of 5,000 patients, including color fundus photographs of both eyes. It is collected from multiple hospitals and medical centers across China using various fundus cameras resulting in heterogeneous image resolutions and illumination conditions. The training dataset remained unchanged, consisting of 5,281 fundus images, while the ODIR dataset was exclusively reserved for external validation. Despite incorporating advanced regularization techniques such as L2 regularization, dropout layers, and checkpoint saving to mitigate overfitting, Model C achieved an accuracy of only 74% on the ODIR dataset. Class-wise analysis revealed accuracy values of 78.6% for Normal, 71.2% for Glaucoma, and 72.4% for Diabetic Retinopathy. This indicating that the largest performance drop occurred in the Glaucoma class due to inter-device variations and subtle optic disc texture differences. This performance decline is primarily attributed to domain shift arising from variations in imaging devices and illumination as in Figure 14 leading to distributional inconsistencies between the training and testing datasets. Standard image normalization and contrast-limited adaptive histogram equalization (CLAHE) were applied in preprocessing phase. However, no domain adaptation or fine-tuning was performed to preserve experimental isolation between datasets.

**Table 5.** Comparison of the performance between the proposed model and other models.

**Research Paper**	**Datasets**	**Classes No.**	**Method**	**Accuracy**
Automatic detection of 39 fundus diseases and conditions in retinal photographs using deep neural networks^58^.	EYEPACS, Messidor-2, IDRID, REFUGE, PALM, JSIEC, LEDRS	39	DLP.	92.62%
Accurate Diagnosis of Diabetic Retinopathy and Glaucoma Using Retinal Fundus Images Based on Hybrid Features and Genetic Algorithm^60^.	HRF, dataset_2.	2	Decision Tree (DT), Naive Bayes (NB), K-Nearest Neighbor (KNN), Linear Discriminant Analysis (LDA)	96.67% 96.42% 96.29% 92.85%
Eye Diseases Classification Using Deep Learning^61^.	IDRID, JSIEC, MESSIDOR, eyePACS, KAGGLE, REFUGE, ORIGA, BinRushed and ORIGA, STARE	4	ResNet-50, ResNet-101, ResNet-152, EfficientNet-b0, EfficientNet-b4	61% 64% 67% 59% 50%
Classification of Eye Disease from Fundus Images Using EfficientNet^62^.	Kaggle 2015 DR Competition- EYEPACS, DIARETDB0, IDRID, MESSIDOR, MESSIDOR2, Kaggle 2019 APTOS.	10	EfficientNet-B6.	86.00%
The Classification of Eye Diseases from Fundus Images Based on CNN and Pre-trained Models^63^.	IDRID, Ocular Recognition, HRF, Retinal Dataset and DRIVE.	3	CNN, GoogleNet, ResNet 18, VGG 19, AlexNet.	87.4% 92.7%. 92.1% 87.9% 88.9%
An Ensemble of Deep Convolutional Neural Networks Is More Accurate and Reliable than Board-Certified Ophthalmologists at Detecting Multiple Diseases in Retinal Fundus Photographs^64^.	RFMiD	4	Convolutional Ensemble	78.2%
Retinal Disease Diagnosis Using Deep Learning on Ultra-Wide-Field Fundus Images^65^.	in-house dataset comprising 4697 images at the Kangbuk Samsung Hospital Ophthalmology Department	2	ResNet152, Vision Transformer, InceptionResNetV2 RegNet ConVNext	89.17% 87.26% 88.11% 88.54% 89.08%
A Deep Learning-Based Approach for Detecting Diabetic Retinopathy in Retina Images^66^.	DRD, Messidor2, IDRiD, RFMiD.	5	CNN	79.96% 94.75% 96.74% 89.10%
A Deep Learning Framework for the Early Detection of Multi-Retinal Diseases^67^.	RFMiD	4	CNN	89.81%
**Proposed Model (Model C)**	**IDRID,** **JSEIC,** **Messidor,** **Kaggle,** **REFUGE,** **ORIGA,** **BinRushed,** **eyePACS,** **JSIEC,** **STARE.**	**3**	**Features Extraction by MobileNetV2, DWT and DenseNet121 with ANN Classifier**	**98.2%**

## Conclusion & Future work

The integration of deep learning algorithms into medical diagnostics represents a significant advancement in the detection and classification of retinal diseases, such as diabetic retinopathy and glaucoma. Traditional diagnostic methods, which often rely on subjective interpretations by clinicians, are prone to variability and potential errors. These limitations are being effectively addressed by the rapid, precise, and scalable analysis capabilities of deep learning technologies. This research demonstrates the potential of deep learning models to achieve remarkable accuracy in diagnosing retinal conditions by leveraging a comprehensive dataset of 5,281 color fundus images collected from different public sources. Contrast Limited Adaptive Histogram Equalization (CLAHE) was applied to enhance image contrast, while all images were resized to 512×512 pixels and normalized to a (0, 1) range. This approach ensured a consistent and balanced representation without the need for oversampling or synthetic techniques like SMOTE, which may introduce artifacts in medical imaging. In contrast to CNN and ViT models, the ANN-based classifier adopted in this study provides a computationally lighter and more interpretable alternative capable of maintaining competitive performance. This balance between efficiency, accuracy, and practical applicability supports the rationale for employing ANN as the final classification component in the proposed framework. Four distinct models were evaluated, each employing Artificial Neural Networks (ANNs) in conjunction with various feature extraction and dimensionality reduction techniques. Among these, Model C, which combines the MobileNetV2 and DenseNet121 architectures with Discrete Wavelet Transform (DWT) for feature extraction, emerged as the most effective, achieving a classification accuracy of 98.2%. The other evaluated models, which utilized different combinations of feature extraction methods, also demonstrated strong performance. DenseNet121 achieved an accuracy of 97.4%, MobileNetV2 with DWT achieved 96.6%, and MobileNetV2 alone achieved 98.1%. For future work, it is planned to expand the application of ANNs to classify a broader range of retinal diseases, including age-related macular degeneration (AMD), retinal detachment, macular edema, retinopathy of prematurity (ROP), cardiovascular disease, hypertensive retinopathy, and cataracts, while ensuring balanced datasets for each category in multilabel classification tasks. Moreover, future efforts will focus on improving the model’s generalization performance by evaluating it on unseen datasets with similar retinal color distributions, as well as by exploring color normalization and domain adaptation techniques to enhance model robustness and ensure more consistent generalization across diverse clinical settings. In addition, advanced deep learning techniques^68–71^ provide methodologies that could further enhance the preprocessing and feature extraction stages of the proposed framework. super-resolution and denoising networks^68,69^ could be applied to fundus images to improve vascular and lesion visibility, potentially increasing the discriminative power of features extracted by DenseNet121 and MobileNetV2. Graph-based modeling approaches^70^ could complement the feature fusion strategy by explicitly capturing structural relationships between retinal regions, while generative deep learning methods^71^ could reconstruct subtle pathological patterns, mitigating class imbalance. Integrating these enhancements into the preprocessing or ANN feature fusion pipeline may improve overall classification accuracy and robustness in future work.

## Data Availability

The datasets generated and/or analyzed during the current study are available from the corresponding author on reasonable request.
